# Supplementary material for: APOBEC3 promotes squamous differentiation via IL-1A/AP-1 signaling
Source: Nat Commun. 2025 Dec 14;17:334. doi: 10.1038/s41467-025-67033-8 (PMC12789560; doi:10.1038/s41467-025-67033-8)

Supplementary Fig. 1

A

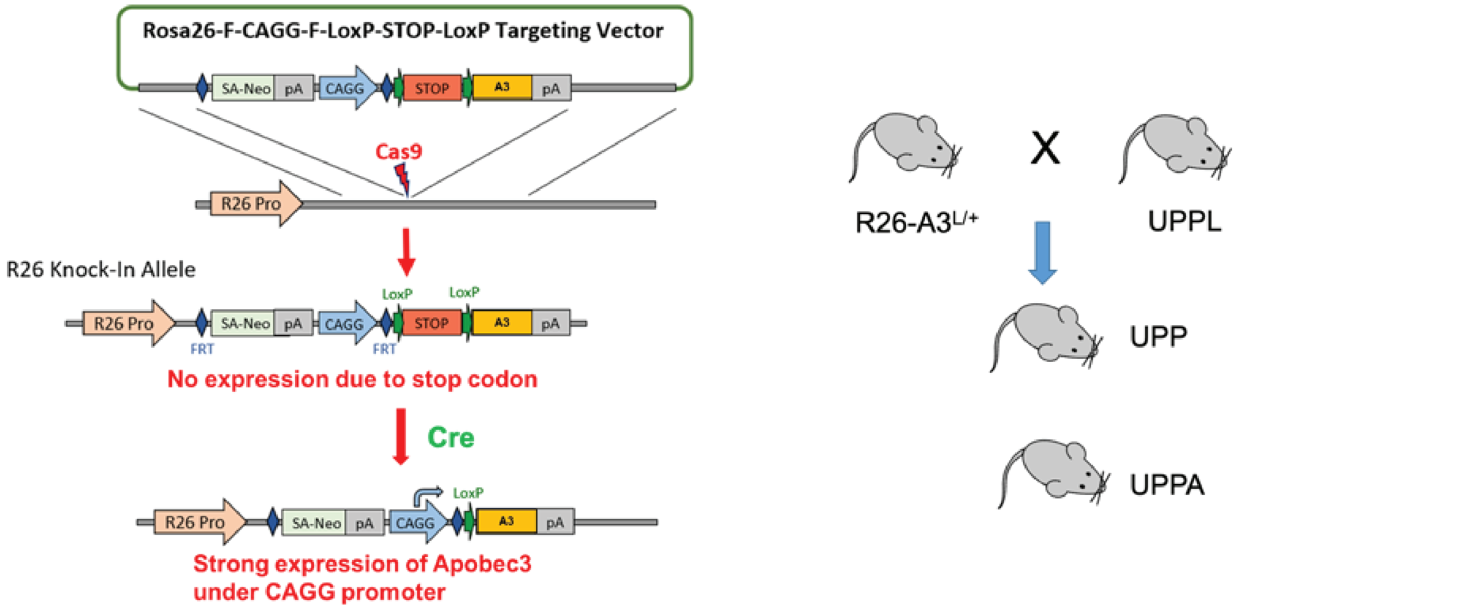

B

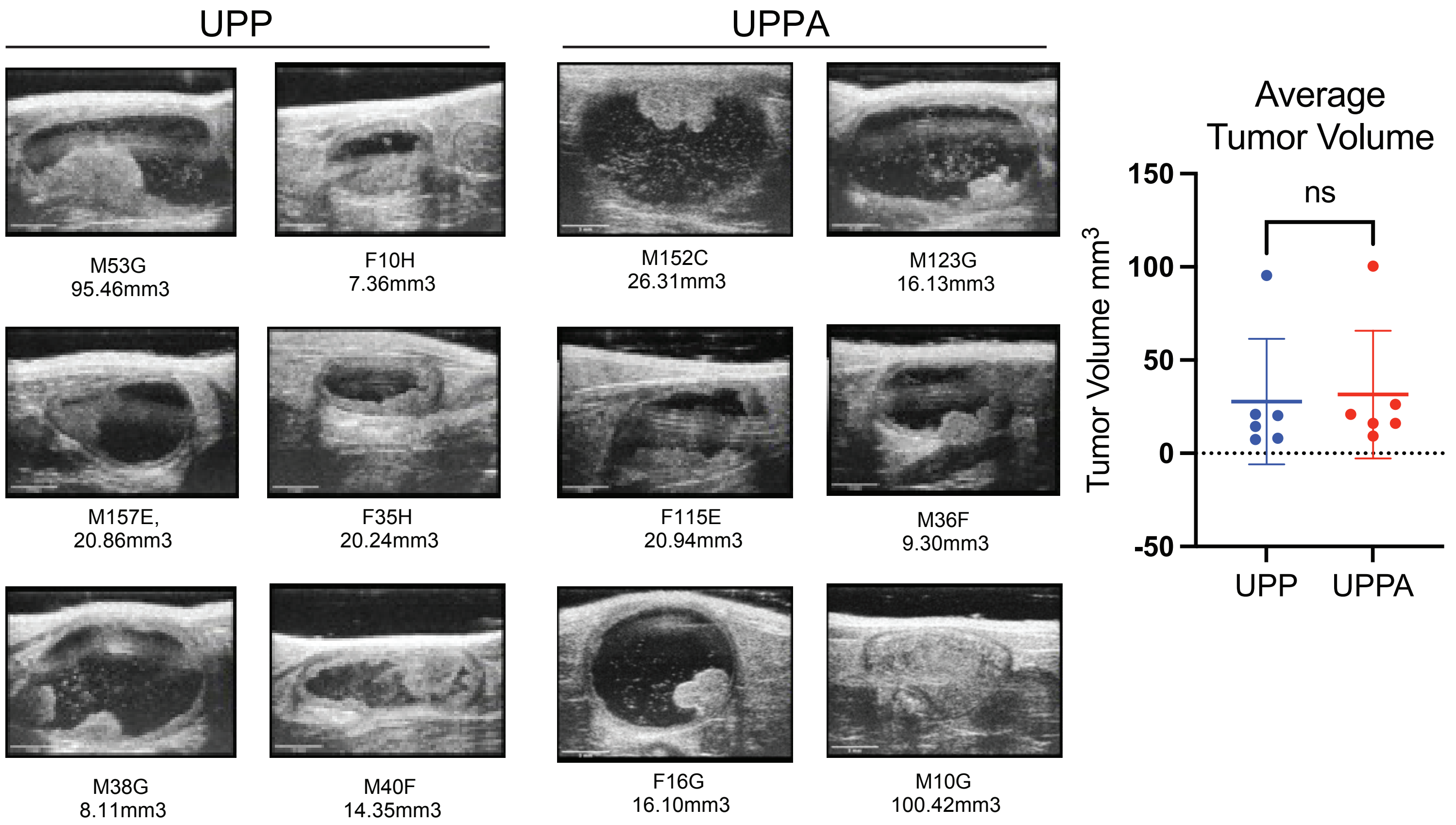

(**A**) Schematic showing targeting of the mA3 cDNA knock-in into the Rosa26 locus and breeding strategy to generate UPP and UPPA mouse models. (**B**) Ultrasound images of UPP and UPPA mice showing bladder tumors and their respective volumes. Scatter plot showing the bladder tumor volume between the UPP and UPPA tumors. Significance was calculated using a two-sided Mann-Whitney test. Data represent mean ± SD (n = 6, biological replicates), ns = not significant.

Supplementary Fig. 2

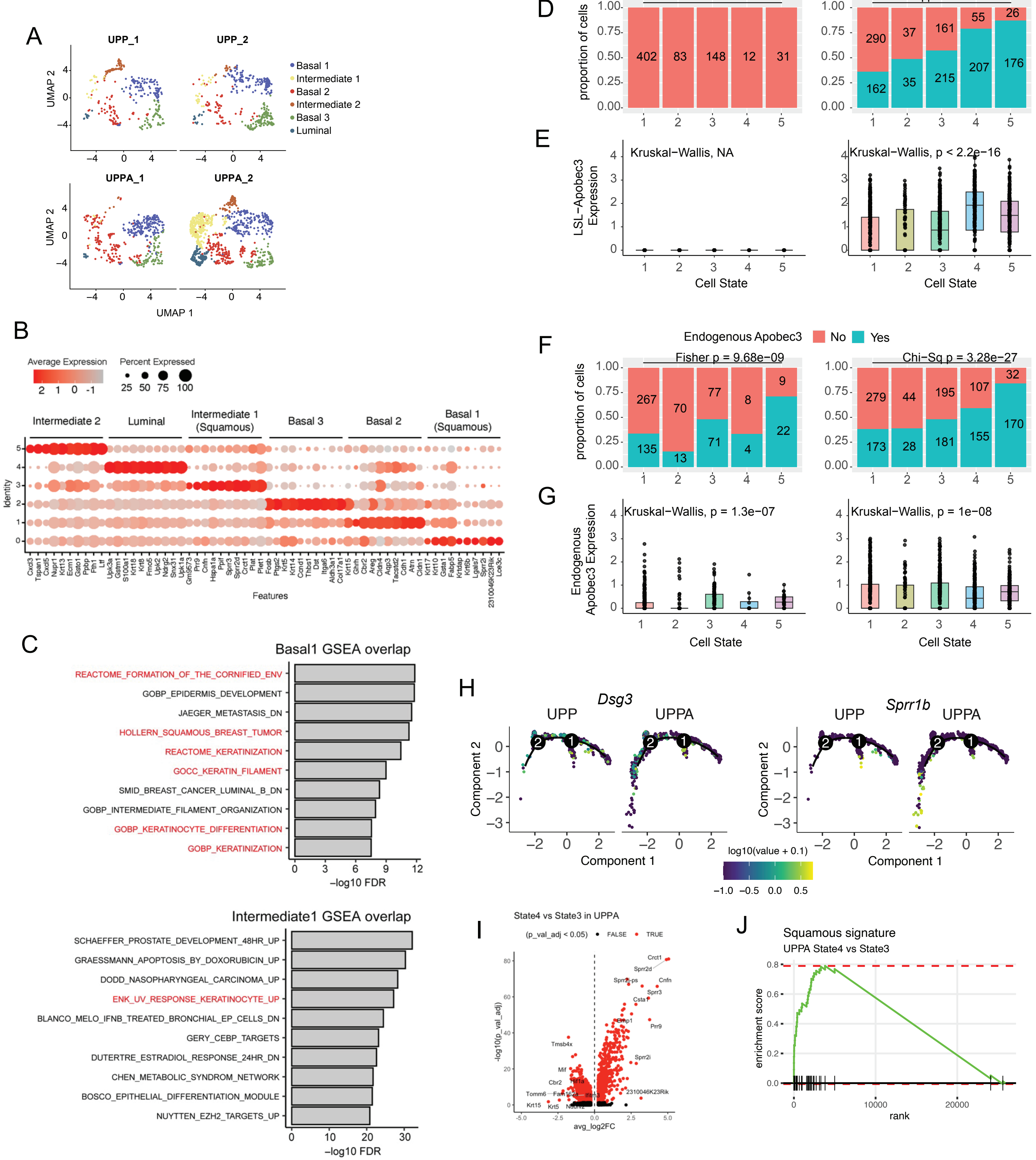

(**A**) Individual UMAP of epithelial cells from scRNAseq of UPP (n=2) and UPPA (n=2) tumors showing six clusters of epithelial cells by Seurat. (**B**) Expression of most differentially expressed genes between the six clusters of epithelial cells. The size of the dots represents the percentage of cells that expressed the genes in the cluster and the color intensity represents the average expression of the genes. (**C**) Barplots of GSEA analysis of Basal 1 and Intermediate 1 -vs- others epithelial cell subpopulation showing the prevalence of pathways. **(D)** Proportion of cells by cell state that express LSL-Apobec3 and **(E)** the expression by cell state of LSL-Apobec3 within cells from UPP and UPPA tumors. **(F)** Proportion of cells by cell state that express endogenous Apobec3 and **(G)** the expression by cell state of Apobec3 within cells from UPP and UPPA tumors. (**H**) Expression of indicated genes in the trajectory inference analysis of epithelial cell populations in UPPA and UPP primary tumors. **(I)** volcano plot of differentially expressed genes between CellState4 and CellState3 in UPPA tumors. **(J)** GSEA plot of our squamous signature comparing CellState4 and CellState3 in UPPA tumors.

Supplementary Fig. 3

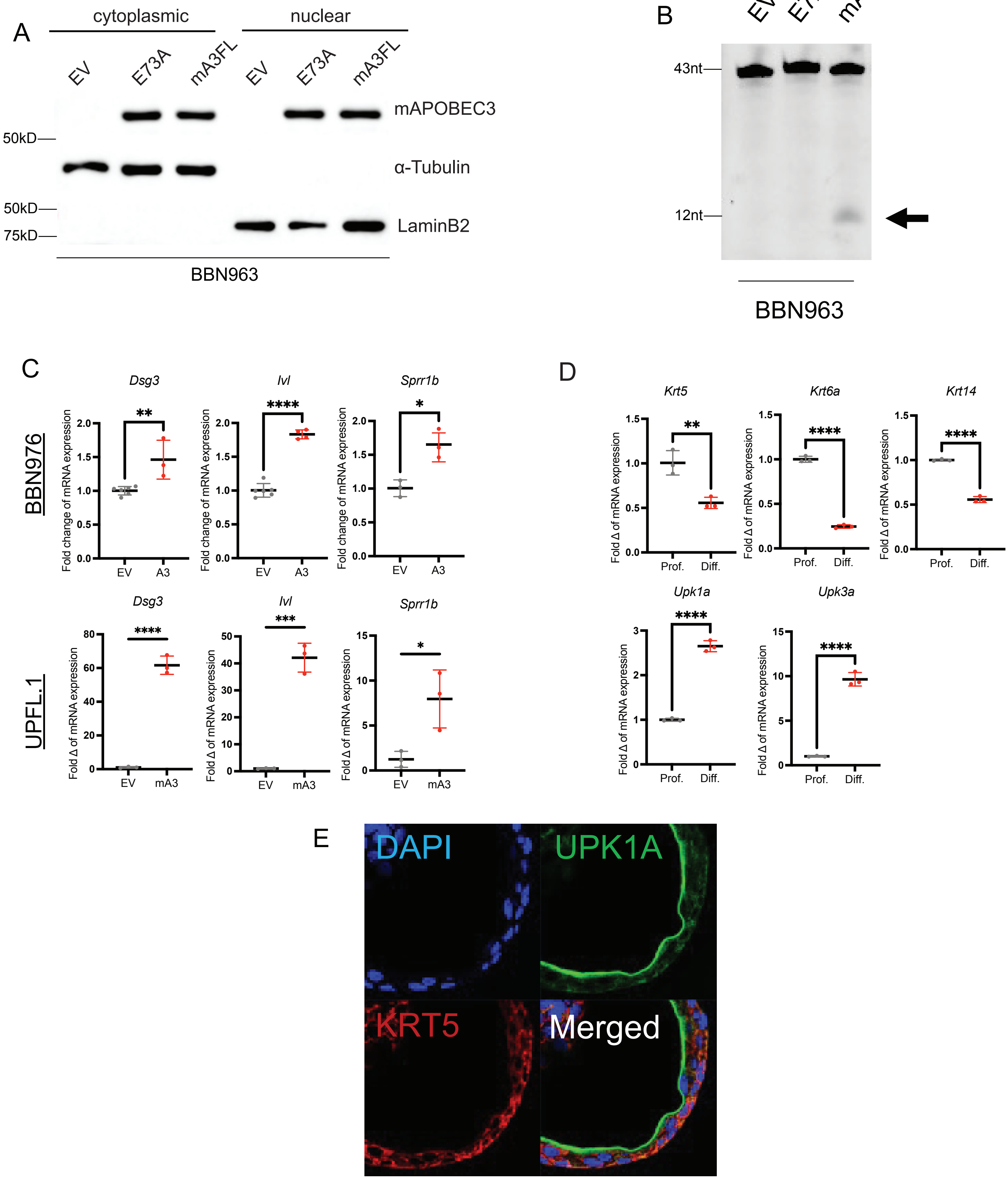

(A) Western blots of nuclear and cytoplasmic cell fractions from BBN963-EV, BBN963-E73A, and BBN976-mA3 treated with doxycycline for 7 days blotted with indicated antibodies **(B)** Cytosine deaminase assay of whole cell lysates from BBN963-EV, BBN963-E73A, and BBN963-mA3 treated with doxycycline for 7days. Black arrow indicates cleaved substrate product, resulting from uracil excision. (**C**) Scatter plots showing the expression of squamous markers (Dsg3, Ivl, and Sprr1b) in BBN976-EV, BBN976-mA3, UPFL.1-EV, and UPFL.1-mA3 cells after 7 days of doxycycline treatment. Significance was calculated using a two-sided unpaired t-test. Data represent mean ± SD (n = 3, technical replicates). * $p < 0.05$, ** $p < 0.01$, *** $p < 0.001$, **** $p < 0.0001$. (**D**) Scatter plots showing the expression of basal and luminal markers in organoids cultured in proliferation (Prof.) or differentiation (Diff) media. Significance was calculated using a two-sided unpaired t-test. Data represent mean ± SD (n = 3, technical replicates). ** $p < 0.01$, **** $p < 0.0001$. (**E**) Confocal immunofluorescence images of organoids cultures in differentiation media labelled with indicated antibodies.

Supplementary Fig. 4

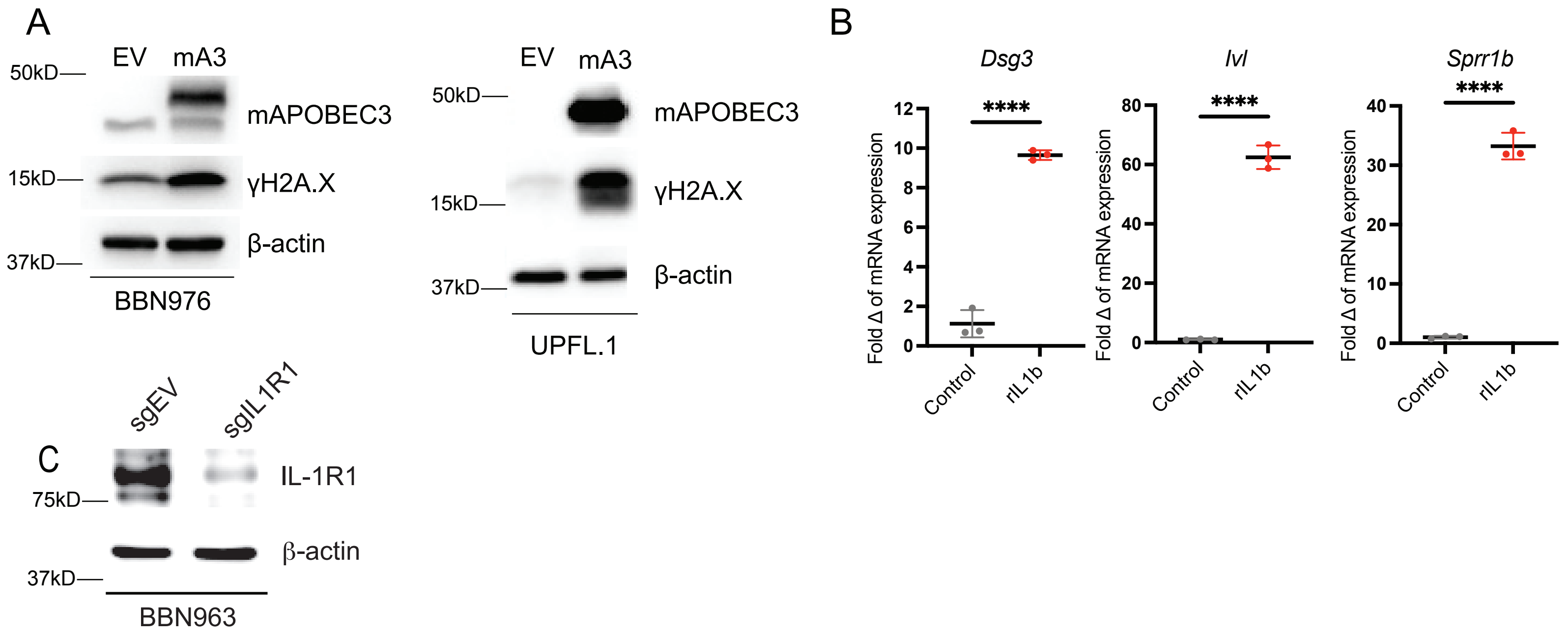

(A) Western blots of whole cell lysates from BBN976-EV, BBN976-mA3, UPFL.1-EV, and UPFL.1-mA3 treated with doxycycline for 7 days blotted with indicated antibodies. **(B)** Scatter plots showing the expression of squamous markers in parental BBN963 cells treated with recombinant IL1B for 72 hours. Significance was calculated using a two-sided unpaired t-test. Data represent mean ± SD (n = 3, technical replicates). **** $p < 0.0001$. **(C)** Western blots of whole cell lysates from BBN963 cells transducted with sgEV or sgIL1R1 and blotted to with the indicated antibodies.

Supplementary Fig. 5

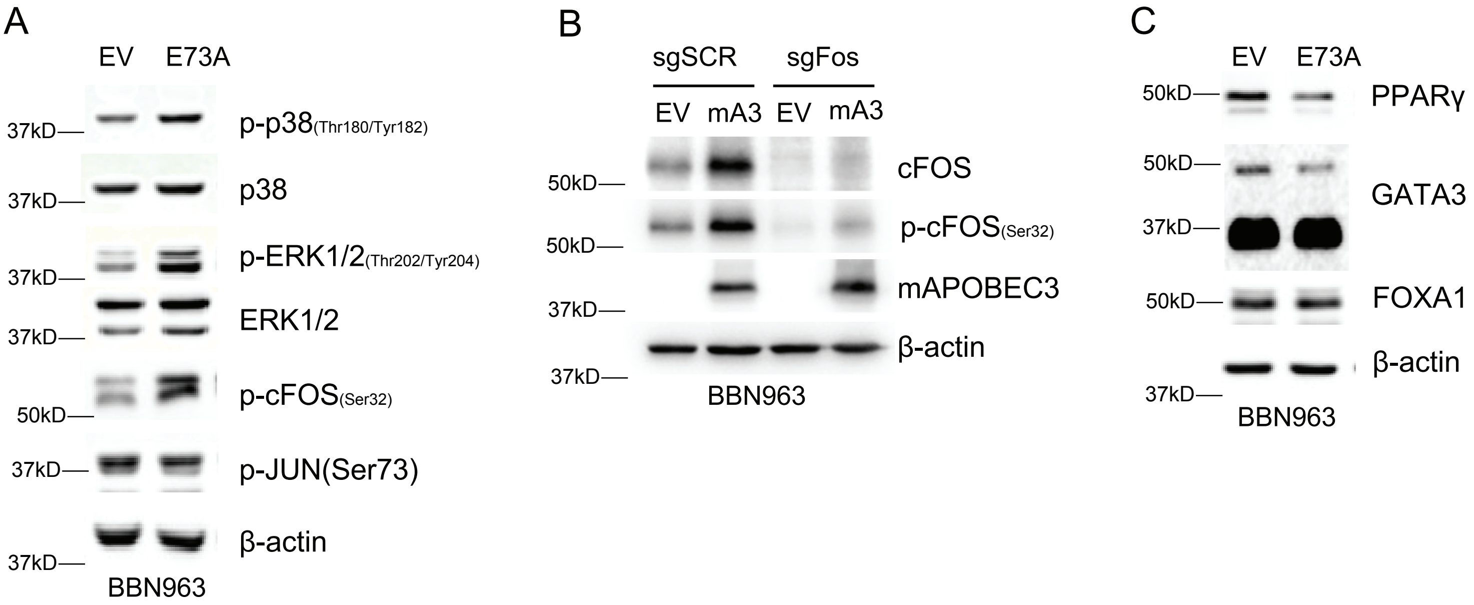

(**A**)Western blots of whole cell lysate from BBN963-EV and BBN963-E73A cells treated with doxycycline for 7 days blotted for indicated antibodies.(**B**) Western blots of whole cell lysates from BBN963-SCR and BBN963-mA3 cells with Fos knockout after 2 days of doxycycline treatment showing knockout of cFos. (**C**) Western blots of whole cell lysate from BBN963-EV and BBN963-E73A cells treated with doxycycline for 7 days blotted for indicated antibodies.

Supplementary Fig. 6

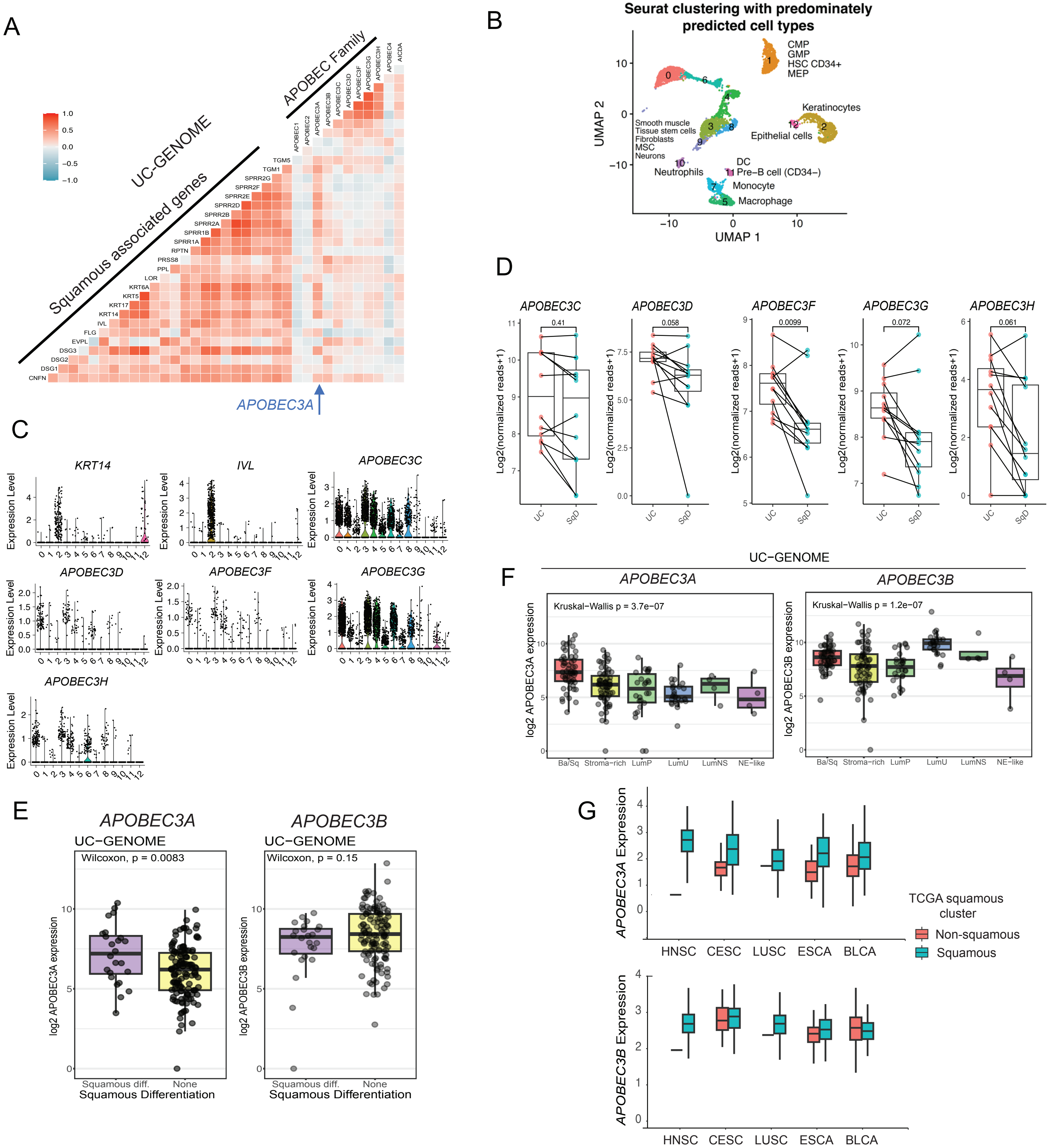

(**A**) Heatmap showing the correlation between expression of human APOBEC genes and squamous markers in UC-GENOME dataset. Blue arrow indicates APOBEC3A. **(B)** UMAP plots of the Warrick scRNA-seq dataset and the cluster based annotation of cell type. **(C)** Violin plots of expression of indicated genes by cluster indicated in (B) from Warrick scRNA-seq dataset. (**D**) Bulk RNAseq counts for areas of UC and squamous differentiation from Warrick et. al. were quantile normalized and log2 transformed. The pseudo-counts were then plotted for APOBEC3C, APOBEC3D, APOBEC3F, APOBEC3G, and APOBEC3H. Wilcoxon p-values are shown above plot. The line plotted in the center of the box represents the median, the hinges represent the upper and lower quartile values, and the whiskers indicate the minimum and maximum values. (**E**) Box plots of human APOBEC3A and APOBEC3B expression in UC-GENOME dataset in tumor samples with or without histologic squamous differentiation. The line plotted in the center of the box represents the median, the hinges represent the upper and lower quartile values, and the whiskers extend 1.5x the interquartile range. **(F)** Box plots of human APOBEC3A and APOBEC3B expression in UC-GENOME dataset in each consensus subtype of bladder cancer. The line plotted in the center of the box represents the median, the hinges represent the upper and lower quartile values, and the whiskers extend 1.5x the interquartile range. (**G**) Box plots comparing the expression of APOBEC3A or APOBEC3B in the TCGA PanSquamous studies. The line plotted in the box represents the median, the hinges represent the upper and lower quartile values, and the whiskers indicate the minimum and maximum values.

Supplementary Fig. 7

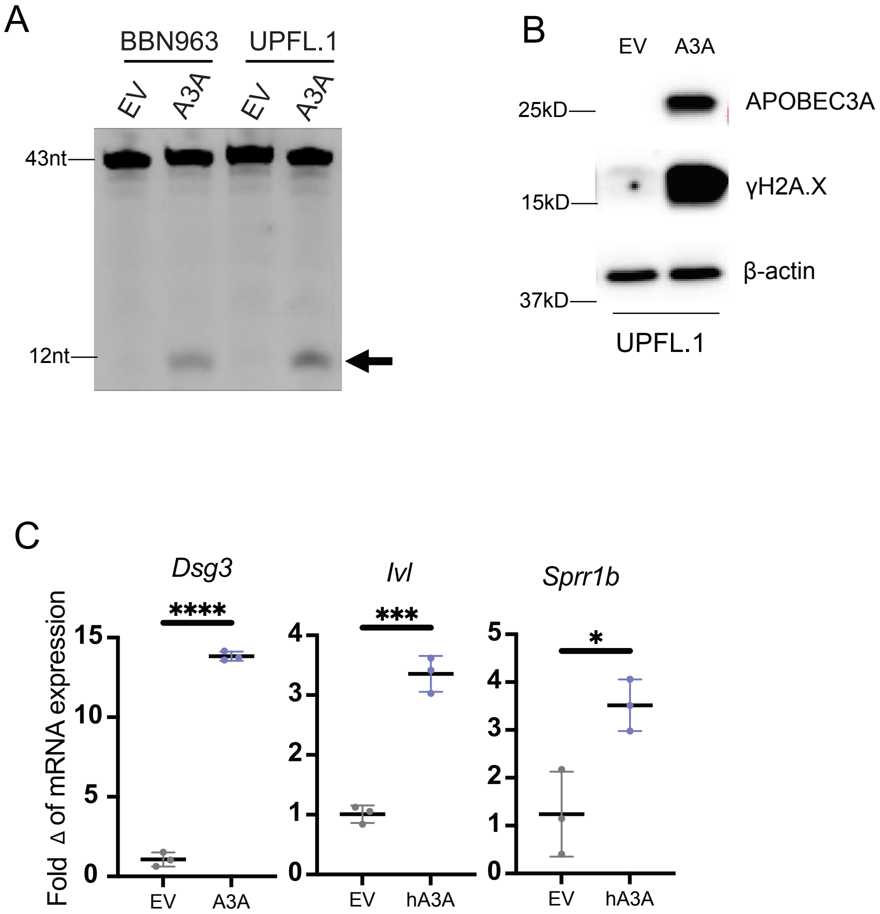

(**A**) Cytosine deaminase assay of whole cell lysates from BBN963-EV and BBN963-A3A treated with doxycycline for 7days. Black arrow indicates cleaved substrate product, resulting from uracil excision. (**B**) Western blots of whole cell lysates from UPFL.1-EV and UPFL.1-A3A treated with doxycycline for 7 days blotted with indicated antibodies. (**C**) Bar plots showing the expression of squamous markers (Dsg3, Ivl, and Sprr1b) in UPFL.1-EV, and UPFL.1-hA3A cells after 7 days of doxycycline treatment. Significance was calculated using a two-sided unpaired t-test. Data represent mean ± SD (n = 3, technical replicates). * $p < 0.05$, *** $p < 0.001$, **** $p < 0.0001$.

Supplementary Table 1: Extent of squamous differentiation within UPP and UPPA tumors

Squamous Differentiation	UPP	UPPA
Extensive (61-99%)	3	6
Moderate (11-60%)	2	4
Minimal (0-10%)	0	1

Supplementary Table 2: Xenium Gene Panel

Gene	Transcript	Gene	Transcript	Gene	Transcript
IVL	ENSG00000163207	TRAC	ENSG00000277734	CD3E	ENSG00000198851
APOBEC3A	ENSG00000128383	ERBB2	ENSG00000141736	DST	ENSG00000151914
SPRR1B	ENSG00000169469	SOX2	ENSG00000181449	CCND1	ENSG00000110092
SERPINB2	ENSG00000197632	MS4A6A	ENSG00000110077	COL5A2	ENSG00000204262
CDKN1A	ENSG00000124762	UBE2C	ENSG00000175063	CD2	ENSG00000116824
S100A12	ENSG00000163221	CD14	ENSG00000170458	KRT20	ENSG00000171431
KRT14	ENSG00000186847	CD74	ENSG00000019582	CAVIN1	ENSG00000177469
FLG	ENSG00000143631	CD4	ENSG00000010610	STEAP4	ENSG00000127954
NECTIN4	ENSG00000143217	CTSK	ENSG00000143387	HAVCR2	ENSG00000135077
STC1	ENSG00000159167	HLA-DRA	ENSG00000204287	EDN1	ENSG00000078401
MTRNR2L11	ENSG00000270188	CCL5	ENSG00000271503	CFB	ENSG00000243649
TCIM	ENSG00000176907	PPARG	ENSG00000132170	CD3D	ENSG00000167286
SERPINB3	ENSG00000057149	CENPF	ENSG00000117724	CD93	ENSG00000125810
MALL	ENSG00000144063	VIM	ENSG00000026025	CD163	ENSG00000177575
PRDM1	ENSG00000057657	MKI67	ENSG00000148773	MMP14	ENSG00000157227
GPRC5A	ENSG00000013588	CCNB2	ENSG00000157456	CD34	ENSG00000174059
C15orf48	ENSG00000166920	TNC	ENSG00000041982	MPEG1	ENSG00000197629
SLC2A1	ENSG00000117394	FGL2	ENSG00000127951	FAS	ENSG00000026103
VEGFA	ENSG00000112715	TP53	ENSG00000141510	CD8A	ENSG00000153563
ALDH1A3	ENSG00000184254	AGR3	ENSG00000173467	RB1	ENSG00000139687
BASP1	ENSG00000176788	SHH	ENSG00000164690	MET	ENSG00000105976
KRT6A	ENSG00000205420	ACTA2	ENSG00000107796	POLD1	ENSG00000062822
DSG3	ENSG00000134757	CDK1	ENSG00000170312	AKT1	ENSG00000142208
TGFB1	ENSG00000105329	TCF4	ENSG00000196628	PPP1R1B	ENSG00000131771
LY6D	ENSG00000167656	FOXM1	ENSG00000111206	GNLY	ENSG00000115523
CTNNB1	ENSG00000168036	CXCL9	ENSG00000138755	VWF	ENSG00000110799
CD44	ENSG00000026508	TOP2A	ENSG00000131747	ADGRL4	ENSG00000162618
TP63	ENSG00000073282	THY1	ENSG00000154096	MLPH	ENSG00000115648
MCF2L	ENSG00000126217	EPCAM	ENSG00000119888	PLA2G7	ENSG00000146070
PARP1	ENSG00000143799	PTGDS	ENSG00000107317	VCAM1	ENSG00000162692
AQP3	ENSG00000165272	CYP4B1	ENSG00000142973	PDGFRA	ENSG00000134853
GPX2	ENSG00000176153	PECAM1	ENSG00000261371	FOXA1	ENSG00000129514
SP1	ENSG00000185591	PDGFRB	ENSG00000113721	CXCR4	ENSG00000121966
PTPRC	ENSG00000081237	APCDD1	ENSG00000154856	WNT5A	ENSG00000114251
KRT7	ENSG00000135480	TGFBR3	ENSG00000069702	FHL2	ENSG00000115641
PTEN	ENSG00000171862	UPK3B	ENSG00000243566	CLEC14A	ENSG00000176435
LAMP3	ENSG00000078081	TBX3	ENSG00000135111	NKG7	ENSG00000105374
CXCL10	ENSG00000169245	TENT5C	ENSG00000183508	PLCG2	ENSG00000197943
ARID1A	ENSG00000117713	FBLN1	ENSG00000077942	STAG2	ENSG00000101972
GPC1	ENSG00000063660	MZB1	ENSG00000170476	NPDC1	ENSG00000107281
STAT1	ENSG00000115415	GATA3	ENSG00000107485	COL17A1	ENSG00000065618
KDM6A	ENSG00000147050	MEST	ENSG00000106484	CD247	ENSG00000198821
RXRA	ENSG00000186350	FGFR3	ENSG00000068078	SLAMF7	ENSG00000026751
CYTIP	ENSG00000115165	CPA3	ENSG00000163751	PMP22	ENSG00000109099
KMT2D	ENSG00000167548	ADAM28	ENSG00000042980	PRF1	ENSG00000180644
PADI3	ENSG00000142619	AIF1	ENSG00000204472	HIF1A	ENSG00000100644
RBPMS	ENSG00000157110	MEF2C	ENSG00000081189	COCH	ENSG00000100473
PTN	ENSG00000105894	MTOR	ENSG00000198793	RARA	ENSG00000131759
FKBP11	ENSG00000134285	SNCG	ENSG00000173267	MS4A4A	ENSG00000110079
PCNA	ENSG00000132646	GZMA	ENSG00000145649	MS4A1	ENSG00000156738

Gene	Transcript	Gene	Transcript	Gene	Transcript
HLA-DQB2	ENSG00000232629	AR	ENSG00000169083	SERPINB9	ENSG00000170542
RARB	ENSG00000077092	PCOLCE	ENSG00000106333	CCR7	ENSG00000126353
CTLA4	ENSG00000163599	LTBP2	ENSG00000119681	TMC5	ENSG00000103534
GPC3	ENSG00000147257	MSN	ENSG00000147065	EGFR	ENSG00000146648
EVPL	ENSG00000167880	UPK2	ENSG00000110375	ACTG2	ENSG00000163017
BANK1	ENSG00000153064	CAPN8	ENSG00000203697	ACE2	ENSG00000130234
RIDA	ENSG00000132541	GNG11	ENSG00000127920	CSF2RA	ENSG00000198223
EGFL7	ENSG00000172889	PIK3CA	ENSG00000121879	RTKN2	ENSG00000182010
SLC26A2	ENSG00000155850	VCAN	ENSG00000038427	EDNRB	ENSG00000136160
CRISPLD2	ENSG00000103196	ELF5	ENSG00000135374	ETV1	ENSG00000006468
FCGR3A	ENSG00000203747	CAVIN2	ENSG00000168497	CD1E	ENSG00000158488
GZMB	ENSG00000100453	NTAN1	ENSG00000157045	IGF1	ENSG00000017427
TNFRSF17	ENSG00000048462	SFTA2	ENSG00000196260	TNFRSF13B	ENSG00000240505
LAG3	ENSG00000089692	HES4	ENSG00000188290	CD19	ENSG00000177455
MYLK	ENSG00000065534	KCNMA1	ENSG00000156113	KRT5	ENSG00000186081
SMYD2	ENSG00000143499	ADH1C	ENSG00000248144	RAPGEF3	ENSG00000079337
KLRD1	ENSG00000134539	DUSP2	ENSG00000158050	PLIN4	ENSG00000167676
CD27	ENSG00000139193	RND1	ENSG00000172602	TMEM52B	ENSG00000165685
VSIG4	ENSG00000155659	TFPI	ENSG00000003436	TM4SF18	ENSG00000163762
CD40	ENSG00000101017	BCL2L11	ENSG00000153094	MDM2	ENSG00000135679
C1orf162	ENSG00000143110	SELL	ENSG00000188404	ASPN	ENSG00000106819
ITGAX	ENSG00000140678	FGFBP1	ENSG00000137440	KLRC1	ENSG00000134545
STAT3	ENSG00000168610	MNDA	ENSG00000163563	TAC1	ENSG00000006128
CD79A	ENSG00000105369	KIAA1217	ENSG00000120549	NPR3	ENSG00000113389
FBLN5	ENSG00000140092	ANGPT2	ENSG00000091879	IL1RL1	ENSG00000115602
GZMK	ENSG00000113088	SMAD3	ENSG00000166949	CD28	ENSG00000178562
CAV1	ENSG00000105974	ITGA11	ENSG00000137809	CLCA2	ENSG00000137975
CD86	ENSG00000114013	FGFR1	ENSG00000077782	CD68	ENSG00000129226
APOLD1	ENSG00000178878	TNFRSF9	ENSG00000049249	BICD1	ENSG00000151746
SNAI2	ENSG00000019549	LIF	ENSG00000128342	SNCA	ENSG00000145335
VWA5A	ENSG00000110002	INMT	ENSG00000241644	LY86	ENSG00000112799
PDPN	ENSG00000162493	LILRB4	ENSG00000186818	MCEMP1	ENSG00000183019
IRF8	ENSG00000140968	FOXJ1	ENSG00000129654	IL2RA	ENSG00000134460
FBN1	ENSG00000166147	ADGRE1	ENSG00000174837	CFTR	ENSG00000001626
FCGR1A	ENSG00000150337	ADAMTS1	ENSG00000154734	TFF2	ENSG00000160181
ECSCR	ENSG00000249751	DES	ENSG00000175084	GPR183	ENSG00000169508
CCR2	ENSG00000121807	KLRB1	ENSG00000111796	GLIPR1	ENSG00000139278
KIT	ENSG00000157404	HPGDS	ENSG00000163106	MYC	ENSG00000136997
RAMP2	ENSG00000131477	MMRN2	ENSG00000173269	DMBT1	ENSG00000187908
MS4A2	ENSG00000149534	LILRB2	ENSG00000131042	TWIST1	ENSG00000122691
THBS2	ENSG00000186340	TIMP4	ENSG00000157150	FAP	ENSG00000078098
IGSF6	ENSG00000140749	FOXP3	ENSG00000049768	THAP2	ENSG00000173451
CDH1	ENSG00000039068	SPI1	ENSG00000066336	IL3RA	ENSG00000185291
CD83	ENSG00000112149	DeltaNp63	DeltaNp63	SLC18A2	ENSG00000165646
IL7R	ENSG00000168685	SMIM24	ENSG00000095932	HAMP	ENSG00000105697
GATM	ENSG00000171766	SPDEF	ENSG00000124664	MRC1	ENSG00000260314
AQP9	ENSG00000103569	SH2D3C	ENSG00000095370	KCNK3	ENSG00000171303
CXCL2	ENSG00000081041	KLK11	ENSG00000167757	CFAP53	ENSG00000172361
IL1R2	ENSG00000115590	C7	ENSG00000112936	TCF15	ENSG00000125878
SFRP2	ENSG00000145423	SLAMF1	ENSG00000117090	HEPACAM2	ENSG00000188175

Gene	Transcript	Gene	Transcript	Gene	Transcript
OPRPN	ENSG00000171199	CCDC39	ENSG00000284862	CYP2A7	ENSG00000198077
PVALB	ENSG00000100362	ASCL1	ENSG00000139352	CXCL6	ENSG00000124875
ASCL3	ENSG00000176009	BMX	ENSG00000102010	PLAC9	ENSG00000189129
TREM2	ENSG00000095970	SOX17	ENSG00000164736	SCARA5	ENSG00000168079
PRG4	ENSG00000116690	NOS2	ENSG00000007171	EHF	ENSG00000135373
JUN	ENSG00000177606	FOXO1	ENSG00000150907	CSF3	ENSG00000108342
ABCA8	ENSG00000141338	SOX18	ENSG00000203883	KNG1	ENSG00000113889
IL6	ENSG00000136244	LILRA4	ENSG00000239961	ABCC11	ENSG00000121270
MMRN1	ENSG00000138722	ADIPOQ	ENSG00000181092	CD70	ENSG00000125726
ESR1	ENSG00000091831	SFRP4	ENSG00000106483	SCGN	ENSG00000079689
FGF2	ENSG00000138685	PLD4	ENSG00000166428	AQP2	ENSG00000167580
AGER	ENSG00000204305	PDCD1	ENSG00000188389	CRTAC1	ENSG00000095713
ARFGEF3	ENSG00000112379	NTN4	ENSG00000074527	IGFL2	ENSG00000204866
UPK1A	ENSG00000105668	MYBPC1	ENSG00000196091	MEDAG	ENSG00000102802
MFAP5	ENSG00000197614	FCER1A	ENSG00000179639	FOXI1	ENSG00000168269
COL11A1	ENSG00000060718	CTSE	ENSG00000196188	SERPINA3	ENSG00000196136
PPP1R1A	ENSG00000135447	TCL1A	ENSG00000100721	ACKR1	ENSG00000213088
GATA2	ENSG00000179348	MMP7	ENSG00000137673	DNAAF1	ENSG00000154099
CLEC10A	ENSG00000132514	SEMA3C	ENSG00000075223	CYP2B6	ENSG00000197408
MYH11	ENSG00000133392	CYP2F1	ENSG00000197446	MLANA	ENSG00000120215
TSPAN19	ENSG00000231738	PROX1	ENSG00000117707	CCL19	ENSG00000172724
CYP3A4	ENSG00000160868	NCAM1	ENSG00000149294	TMEM100	ENSG00000166292
CD69	ENSG00000110848	FGFBP2	ENSG00000137441	CD5L	ENSG00000073754
SLC4A1	ENSG00000004939	CLEC4E	ENSG00000166523	CLIC6	ENSG00000159212
GCG	ENSG00000115263	TNF	ENSG00000232810	RBP5	ENSG00000139194
ICOS	ENSG00000163600	MAF	ENSG00000178573	MAMDC2	ENSG00000165072
S100A1	ENSG00000160678	BCL2	ENSG00000171791	SLC26A3	ENSG00000091138
LYVE1	ENSG00000133800	GEM	ENSG00000164949	SNTN	ENSG00000188817
AQP8	ENSG00000103375	CD274	ENSG00000120217	FXYD2	ENSG00000137731
AMY2A	ENSG00000243480	VSNL1	ENSG00000163032	RERGL	ENSG00000111404
CDKN2A	ENSG00000147889	SELE	ENSG00000007908	HIGD1B	ENSG00000131097
DNASE1L3	ENSG00000163687	ANPEP	ENSG00000166825	C6orf118	ENSG00000112539
PI16	ENSG00000164530	HPX	ENSG00000110169	HEMGN	ENSG00000136929
STC2	ENSG00000113739	NAT8	ENSG00000144035	CLCA1	ENSG00000016490
SLC22A8	ENSG00000149452	TM4SF4	ENSG00000169903	CNIH3	ENSG00000143786
SPIB	ENSG00000269404	PCSK2	ENSG00000125851	CYP1A1	ENSG00000140465
NOX4	ENSG00000086991	CTSG	ENSG00000100448	CR2	ENSG00000117322
FCN1	ENSG00000085265	CCDC78	ENSG00000162004	TAT	ENSG00000198650
GYPA	ENSG00000170180	BTNL9	ENSG00000165810	SST	ENSG00000157005
NR3C1	ENSG00000113580	INS	ENSG00000254647	ERG	ENSG00000157554
SRPX	ENSG00000101955	PCP4	ENSG00000183036	DPEP1	ENSG00000015413
APOBEC3B	ENSG00000179750	APOA5	ENSG00000110243	CCL27	ENSG00000213927
DPT	ENSG00000143196	CD300E	ENSG00000186407	UPK3A	ENSG00000100373
DERL3	ENSG00000099958	CFHR3	ENSG00000116785	GDF15	ENSG00000130513
SNAI1	ENSG00000124216	CD1A	ENSG00000158477	OGN	ENSG00000106809
C5orf46	ENSG00000178776	LGI4	ENSG00000153902	HMGCS2	ENSG00000134240
GHRL	ENSG00000157017	GYPB	ENSG00000250361	VCL	ENSG00000035403
CHGA	ENSG00000100604	AHSP	ENSG00000169877	CHRDL1	ENSG00000101938
GKN2	ENSG00000183607	ALAS2	ENSG00000158578	TMEM174	ENSG00000164325
CD1C	ENSG00000158481	BAMBI	ENSG00000095739	SCGB2A1	ENSG00000124939

Gene	Transcript
LILRA5	ENSG00000187116
CRHBP	ENSG00000145708
C1orf194	ENSG00000179902
FSTL3	ENSG00000070404
FCN2	ENSG00000160339
CA4	ENSG00000167434
MARCO	ENSG00000019169
DSG1	ENSG00000134760
PGR	ENSG00000082175
BBOX1	ENSG00000129151
ADH4	ENSG00000198099
CDH16	ENSG00000166589
CNN1	ENSG00000130176
ITGB5	ENSG00000082781
DIRAS3	ENSG00000162595
GLYATL1	ENSG00000166840
LPL	ENSG00000175445
LGR5	ENSG00000139292
PEBP4	ENSG00000134020
RGS16	ENSG00000143333
CLECL1	ENSG00000184293
PPY	ENSG00000108849
CFHR1	ENSG00000244414
C20orf85	ENSG00000124237
UMOD	ENSG00000169344
ANK2	ENSG00000145362
RETN	ENSG00000104918

Supplemental Table 3: Antibody information

Antibody	Dilution	Source	Cat #	Application	Validation
mApobec3	1:1000	Santa Cruz Biotechnology	sc-390254	WB	Per manufacturer website
γ-H2A.X	1:1000	Cell Signaling Technology	80312	WB	Per manufacturer website
β-actin	1:1000	Cell Signaling Technology	5125	WB	Per manufacturer website
Krt5	1:500	Biolegend	905904	IF	Per manufacturer website
Ivl	1:500	Biolegend	924401	IF	Per manufacturer website
Dsg3	1:500	Santa Cruz Biotechnology	sc-53487	IF	Per manufacturer website
Krt6a	1:500	Biolegend	905701	IF	Per manufacturer website
p-p38	1:1000	Cell Signaling Technology	4511	WB	Per manufacturer website
p38	1:1000	Cell Signaling Technology	8690	WB	Per manufacturer website
p-ERK1/2	1:1000	Cell Signaling Technology	9106	WB	Per manufacturer website
ERK1/2	1:1000	Cell Signaling Technology	9102	WB	Per manufacturer website
p-cFOS	1:1000	Cell Signaling Technology	5348	WB	Per manufacturer website
p-JUN	1:1000	Cell Signaling Technology	3270	WB	Per manufacturer website
p-IKKα/β	1:1000	Cell Signaling Technology	2697	WB	Per manufacturer website
IKKβ	1:1000	Cell Signaling Technology	8943	WB	Per manufacturer website
p-p65	1:1000	Cell Signaling Technology	3033	WB	Per manufacturer website
p65	1:1000	Cell Signaling Technology	8242	WB	Per manufacturer website
TNFAIP3	1:1000	Cell Signaling Technology	5630	WB	Per manufacturer website
PPARγ	1:1000	Cell Signaling Technology	2430	WB	Per manufacturer website
GATA-3	1:1000	Cell Signaling Technology	5852	WB	Per manufacturer website
FOXA1	1:1000	Abcam	ab173287	WB	Per manufacturer website
IL-1α	2μg/mL	Biolegend	503208	Neutralizing	Per manufacturer website

Uncropped Blots

Supplementary Figure 3A

Western blots of nuclear and cytoplasmic cell fractions from BBN963-EV, BBN963-E73A, and BBN976-mA3 treated with doxycycline for 7 days blotted with indicated antibodies

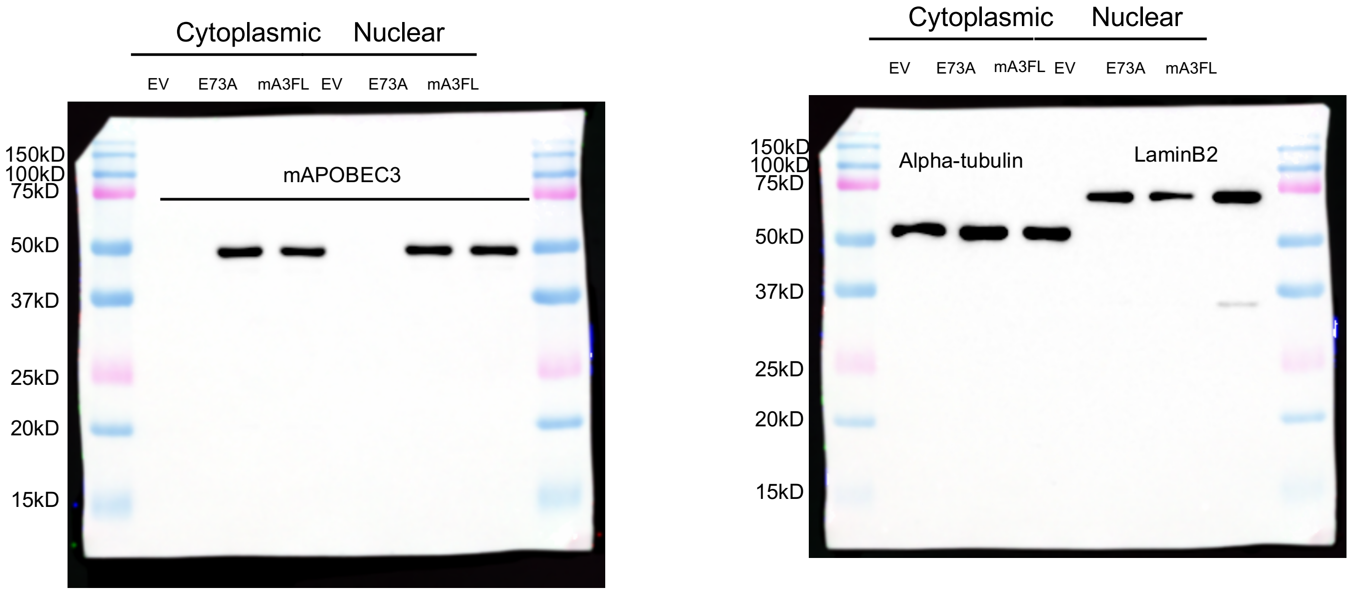

Supplementary Figure 4A

Western blots of whole cell lysates from BBN976-EV, BBN976-mA3, UPFL.1-EV, and UPFL.1-mA3 treated with doxycycline for 7 days blotted with indicated antibodies.

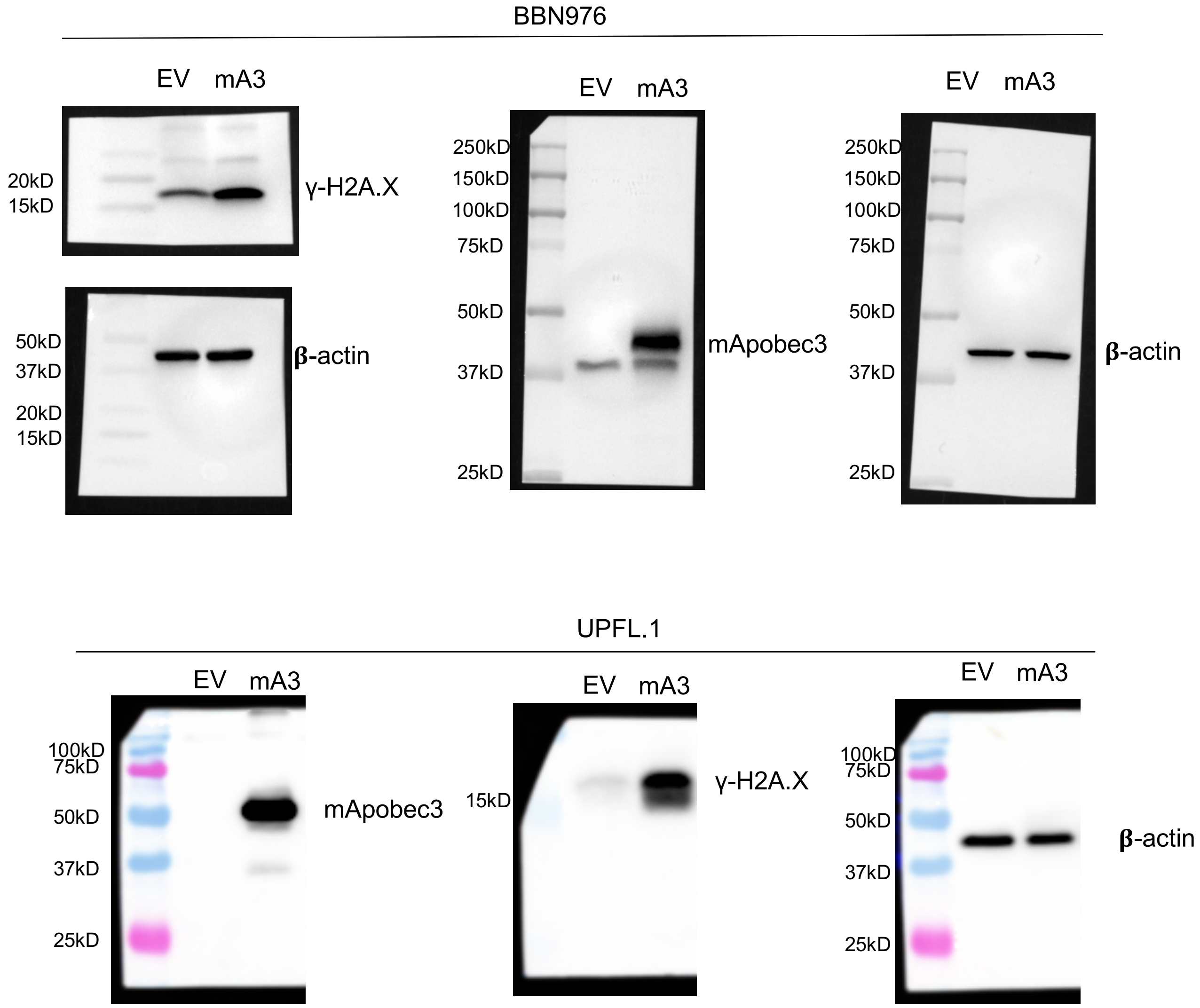

Supplementary Figure 4C

Western blots of whole cell lysates from BBN963 cells transducted with sgEV or sgIL1R1 and blotted to with the indicated antibodies.

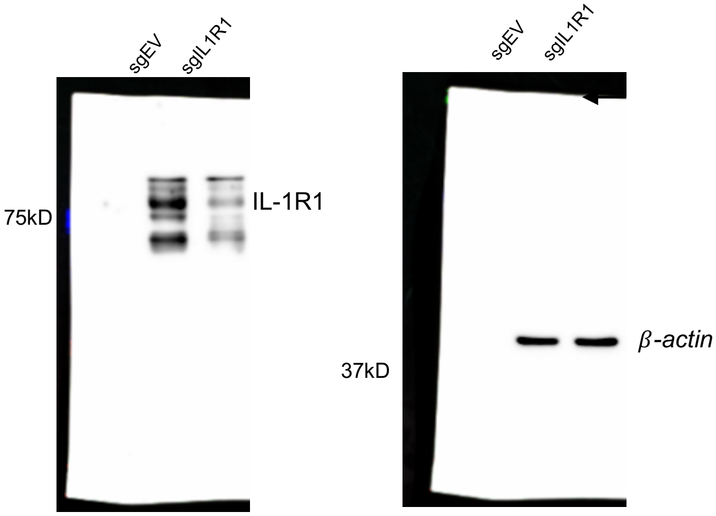

Supplementary Figure 5A

Western blots of whole cell lysate from BBN963-EV and BBN963-E73A cells treated with doxycycline for 7 days blotted for indicated antibodies.

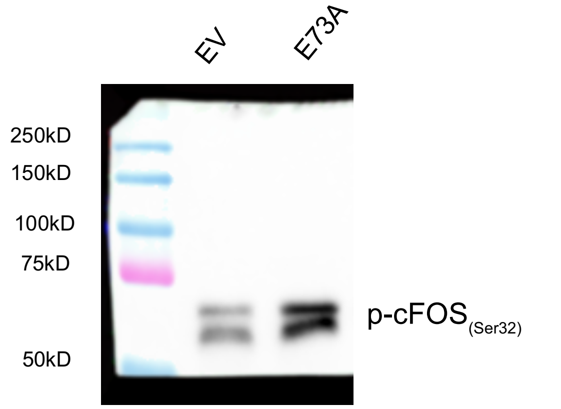

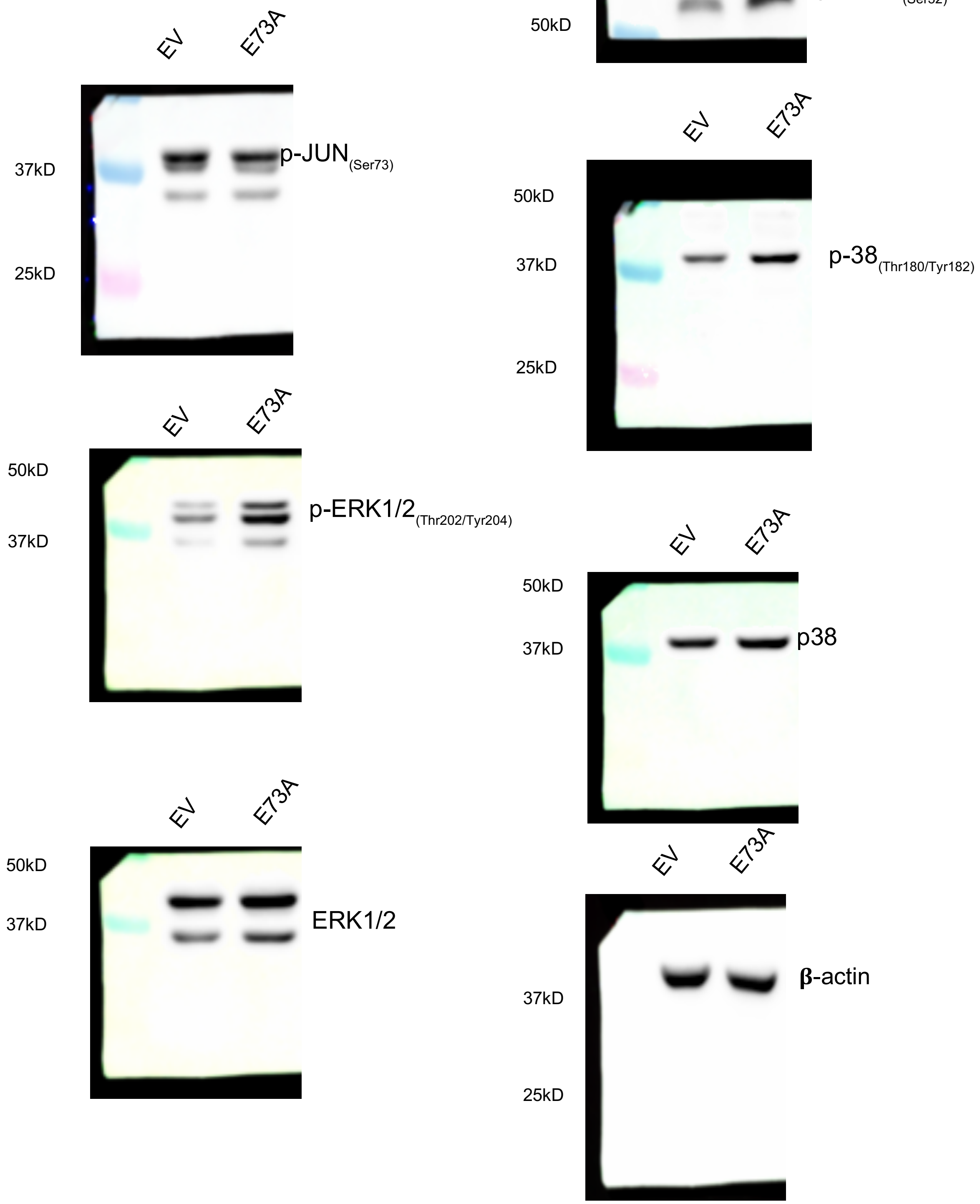

Supplementary Figure 5B

Western blots of whole cell lysates from BBN963-SCR and BBN963-mA3 cells with Fos knockout after 2 days of doxycycline treatment

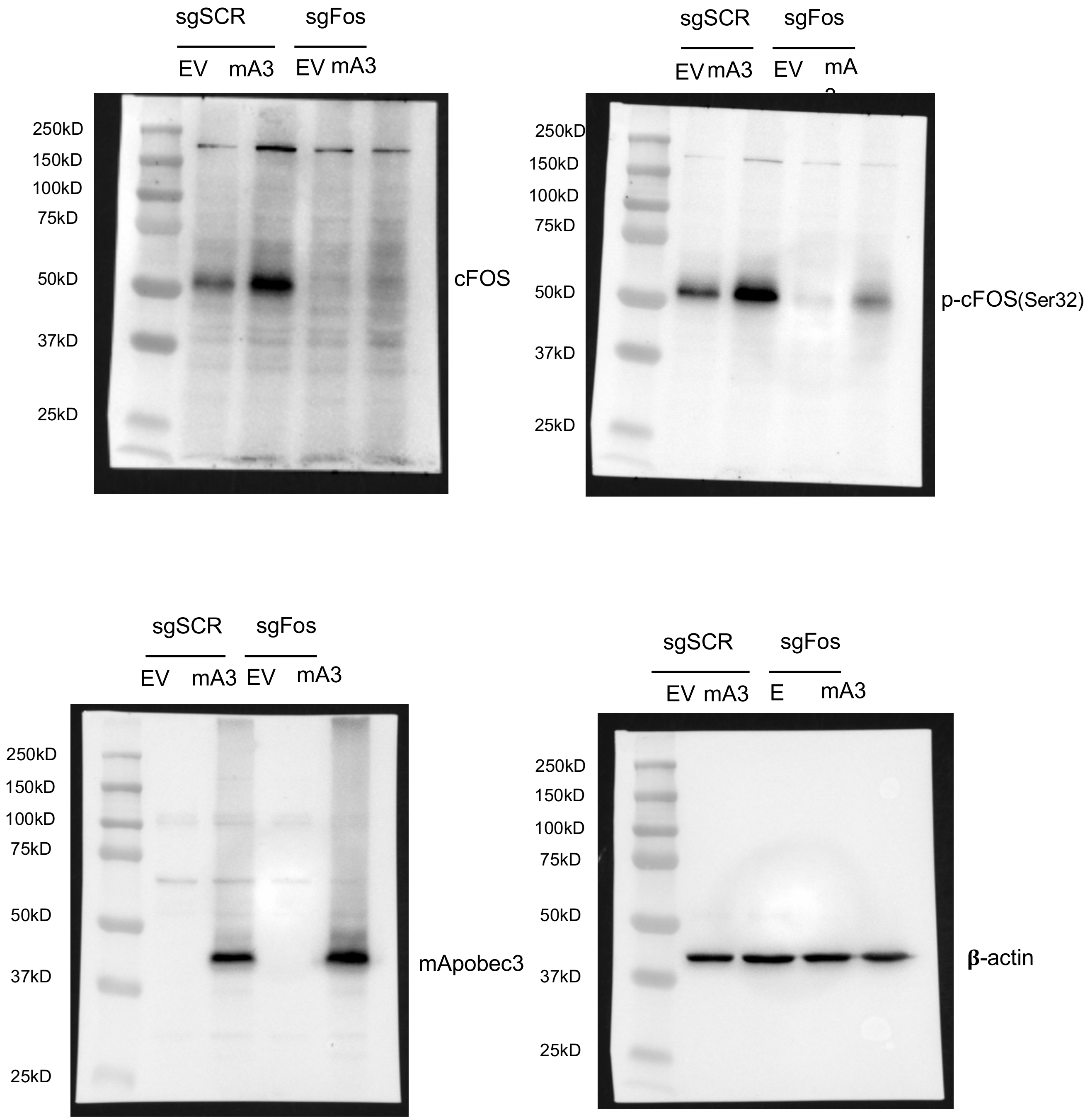

Supplementary Figure 5C

Western blots of whole cell lysate from BBN963-EV and BBN963-E73A cells treated with doxycycline for 7 days blotted for indicated antibodies.

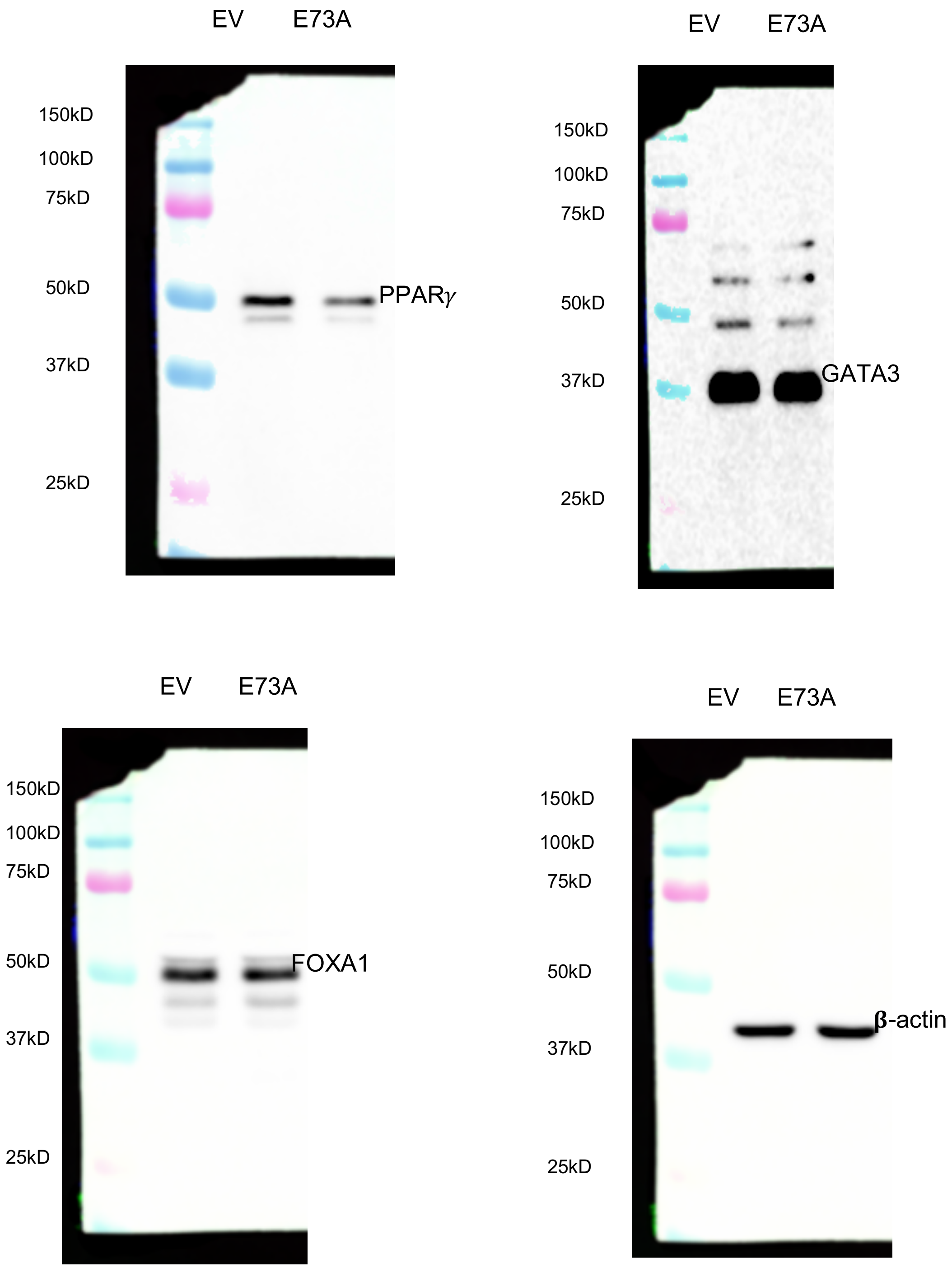

Supplementary Figure 7A

Cytosine deaminase assay of whole cell lysates from BBN963-EV and BBN963-A3A treated

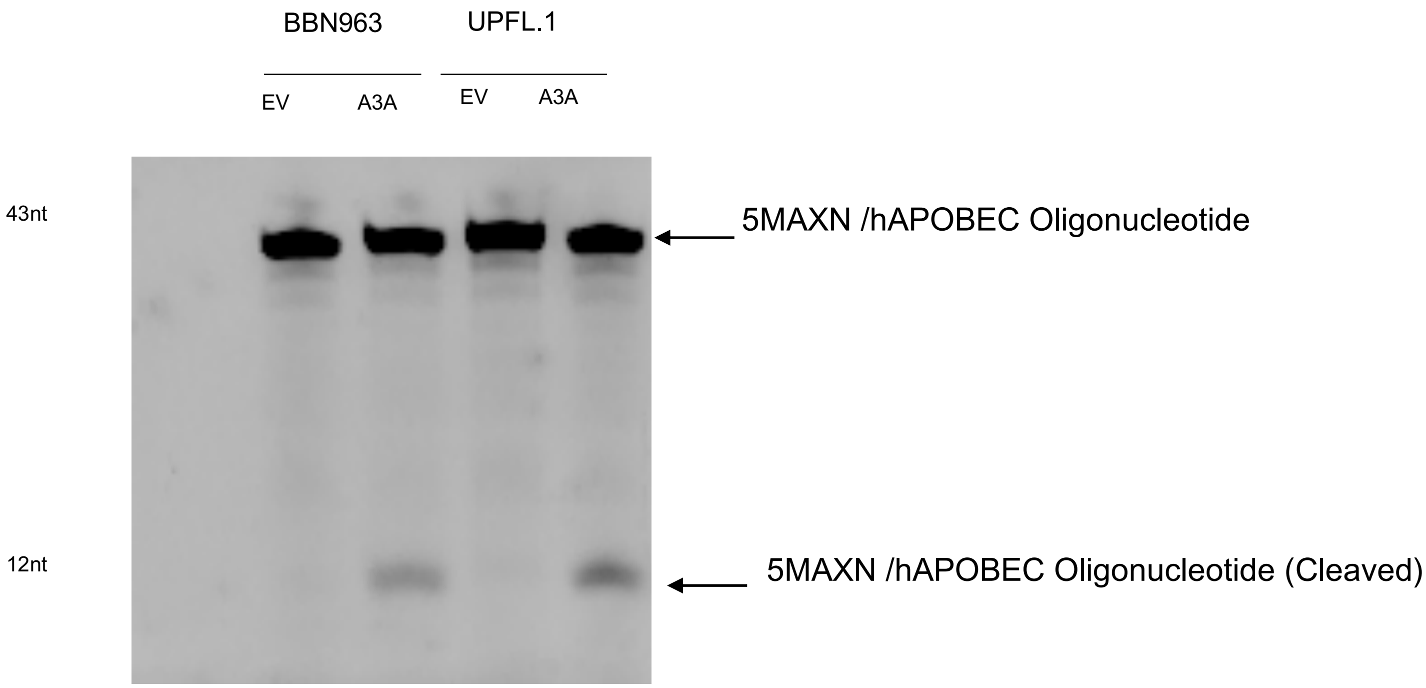

Supplementary Figure 7B

Western blots of whole cell lysates from UPFL.1-EV and UPFL.1-A3A treated with doxycycline for 7 days blotted with indicated antibodies with doxycycline for 7days.

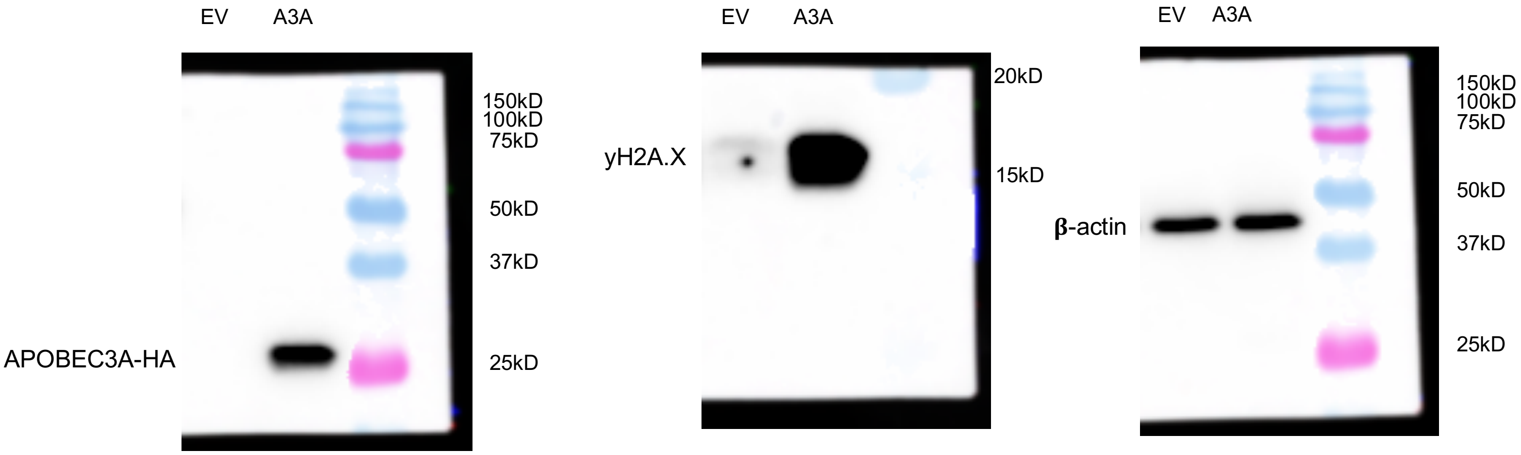

Supplement: Supplementary file 1 — Supplementary Information [file 41467_2025_67033_MOESM1_ESM.pdf]
