# Supplementary material for: APOBEC3 promotes squamous differentiation via IL-1A/AP-1 signaling
Source: Nat Commun. 2025 Dec 14;17:334. doi: 10.1038/s41467-025-67033-8 (PMC12789560; doi:10.1038/s41467-025-67033-8)
Supplement: Supplementary file 3 — Reporting Summary [file 41467_2025_67033_MOESM3_ESM.pdf]

nature portfolio

Corresponding author(s): William Y. Kim

Last updated by author(s): 6/18/2025

Reporting Summary

Nature Portfolio wishes to improve the reproducibility of the work that we publish. This form provides structure for consistency and transparency in reporting. For further information on Nature Portfolio policies, see our Editorial Policies and the Editorial Policy Checklist.

Statistics

For all statistical analyses, confirm that the following items are present in the figure legend, table legend, main text, or Methods section.

n/a	Confirmed	
☐	☒	The exact sample size (*n*) for each experimental group/condition, given as a discrete number and unit of measurement
☐	☒	A statement on whether measurements were taken from distinct samples or whether the same sample was measured repeatedly
☐	☒	The statistical test(s) used AND whether they are one- or two-sided<br>*Only common tests should be described solely by name; describe more complex techniques in the Methods section.*
☐	☒	A description of all covariates tested
☐	☒	A description of any assumptions or corrections, such as tests of normality and adjustment for multiple comparisons
☐	☒	A full description of the statistical parameters including central tendency (e.g. means) or other basic estimates (e.g. regression coefficient) AND variation (e.g. standard deviation) or associated estimates of uncertainty (e.g. confidence intervals)
☐	☒	For null hypothesis testing, the test statistic (e.g. *F*, *t*, *r*) with confidence intervals, effect sizes, degrees of freedom and *P* value noted<br>*Give P values as exact values whenever suitable.*
☐	☒	For Bayesian analysis, information on the choice of priors and Markov chain Monte Carlo settings
☒	☐	For hierarchical and complex designs, identification of the appropriate level for tests and full reporting of outcomes
☒	☐	Estimates of effect sizes (e.g. Cohen's *d*, Pearson's *r*), indicating how they were calculated

Our web collection on statistics for biologists contains articles on many of the points above.

Software and code

Policy information about availability of computer code

Data collection	RNASeq: Reads were aligned using STAR and quantified using Salmon.<br>scRNA-seq: Reads were processed using Cell Ranger<br>Xenium: Imaging and quantification was performed using the on board Xenium Software v2.0.1.0

Data analysis

CellRanger 6.1.2
DESeq2 1.50.0
dplyr 1.14
forcats 1.0.1
fsgsea 3.22
ggplot2 4.0.0
monocle 2.4
rstatix 0.7.3
scCustomize 3.2.0
SingleR 3.22
stats 4.5.1
tidyverse 2
Xenium Explorer 3.2
Xenium Software v2.0.1.0
Seurat 5.3.0
R 4.5.0
R Studio 2025.05.0 Build 496

For manuscripts utilizing custom algorithms or software that are central to the research but not yet described in published literature, software must be made available to editors and reviewers. We strongly encourage code deposition in a community repository (e.g. GitHub). See the Nature Portfolio guidelines for submitting code & software for further information.

Data

Policy information about availability of data

All manuscripts must include a data availability statement. This statement should provide the following information, where applicable:

- Accession codes, unique identifiers, or web links for publicly available datasets
- A description of any restrictions on data availability
- For clinical datasets or third party data, please ensure that the statement adheres to our policy

No custom code was generated during the course of this study. The scRNA-sequencing data generated in this study have been deposited in the Gene Expression Omnibus (GEO) repository under accession number GSE237016, [https://www.ncbi.nlm.nih.gov/geo/query/acc.cgi?acc=GSE237016]. Raw sequencing data and spatial transcriptomic data have been deposited in the dbGaP under accession number phs003405.v1.p1, [https://www.ncbi.nlm.nih.gov/projects/gap/cgi-bin/study.cgi?study_id=phs000178.v9.p8].

Research involving human participants, their data, or biological material

Policy information about studies with human participants or human data. See also policy information about sex, gender (identity/presentation), and sexual orientation and race, ethnicity and racism.

Reporting on sex and gender	The deidentified samples were collected under an IRB exempt protocol and the investigators were only privileged to deidentified information that did not include sex and gender.
Reporting on race, ethnicity, or other socially relevant groupings	The deidentified samples were collected under an IRB exempt protocol and the investigators were only privileged to deidentified information that did not include race, ethnicity, or other socially relevant groupings.
Population characteristics	Please see above.
Recruitment	Patients were recruited from outpatient and inpatient areas of the UNC Hospitals. The only eligibility requirement was that they were undergoing a TURBT or cystectomy
Ethics oversight	University of North Carolina Institutional Review Board

Note that full information on the approval of the study protocol must also be provided in the manuscript.

Field-specific reporting

Please select the one below that is the best fit for your research. If you are not sure, read the appropriate sections before making your selection.

☒ Life sciences ☐ Behavioural & social sciences ☐ Ecological, evolutionary & environmental sciences

For a reference copy of the document with all sections, see nature.com/documents/nr-reporting-summary-flat.pdf

Life sciences study design

All studies must disclose on these points even when the disclosure is negative.

Sample size	Mouse study sample size was determined based on our past experience with studies involving assessing survival in GEM models as well as for assays that affect biologic phenotypes.

Data exclusions	No data were excluded from the final analysis.
Replication	All studies were carried out 2 to 3 times. If the study findings validated twice in a row that was considered a positive result. If the study results were inconsistent in the first 2 replicates, the study was repeated a third time to assess which result was true.
Randomization	We did not carry out any therapeutic studies, no randomization was performed.
Blinding	Quantification of squamous histology and histologic characteristics were performed in a blinded fashion. All other experiments were carried out in unblinded fashion as blinding was applicable to the experiment.

Reporting for specific materials, systems and methods

We require information from authors about some types of materials, experimental systems and methods used in many studies. Here, indicate whether each material, system or method listed is relevant to your study. If you are not sure if a list item applies to your research, read the appropriate section before selecting a response.

Materials & experimental systems

n/a	Involved in the study	
☐	☒	Antibodies
☐	☒	Eukaryotic cell lines
☒	☐	Palaeontology and archaeology
☐	☒	Animals and other organisms
☒	☐	Clinical data
☒	☐	Dual use research of concern
☒	☐	Plants

Methods

n/a	Involved in the study	
☒	☐	ChIP-seq
☒	☐	Flow cytometry
☒	☐	MRI-based neuroimaging

Antibodies

Antibodies used

Antibody Source Cat # Application
mApobec3 Santa Cruz Biotechnology sc-390254 WB
γ-H2A.X Cell Signaling Technology 80312 WB
β-actin Cell Signaling Technology 5125 WB
Krt5 Biolegend 905904 IF
Ivl Biolegend 924401 IF
Dsg3 Santa Cruz Biotechnology sc-53487 IF
Krt6a Biolegend 905701 IF
p-p38 Cell Signaling Technology 4511 WB
p38 Cell Signaling Technology 8690 WB
p-ERK1/2 Cell Signaling Technology 9106 WB
ERK1/2 Cell Signaling Technology 9102 WB
p-cFOS Cell Signaling Technology 5348 WB
p-JUN Cell Signaling Technology 3270 WB
p-IKKα/β Cell Signaling Technology 2697 WB
IKKβ Cell Signaling Technology 8943 WB
p-p65 Cell Signaling Technology 3033 WB
p65 Cell Signaling Technology 8242 WB
TNFAIP3 Cell Signaling Technology 5630 WB
PPARγ Cell Signaling Technology 2430 WB
GATA-3 Cell Signaling Technology 5852 WB
FOXA1 Abcam ab173287 WB
IL-1α Biolegend 503208 Neutralizing

Validation

All antibodies have been validated per manufacturer's websites.

Eukaryotic cell lines

Policy information about cell lines and Sex and Gender in Research

Cell line source(s)	BBN963 cells were previously generated within the lab and described in PMID: 29784854. UPFL.1 cells were previously generated within the lab and described in PMID: 38226620. BBN976 cells were the previously derived within the lab, but unpublished. The cells were established from mice of the following sex: BBN963 - Female, UPFL.1 - Male, BBN976 - Male.
Authentication	All cell lines under go yearly STR testing for authentication.
Mycoplasma contamination	All cell lines were tested monthly for Mycoplasma contamination and all tests were negative

Commonly misidentified lines (See ICLAC register)	none used

Animals and other research organisms

Policy information about studies involving animals; ARRIVE guidelines recommended for reporting animal research, and Sex and Gender in Research

Laboratory animals	Rosa26-LSL-mApobec3 mice were generated by the UNC Animal Models Core on a C57BL/6J background (obtained from an in house breeding colony) for the Kim Lab. The Rosa26-LSL-mApobec3 mouse was then crossed to the previously described UPPL model (PMID: PMC6157276) to generate UPP and UPPA mice.<br>As previously reported, the UPPL mice include the following stains/alleles: B6.129P2-Trp53tm1Brn/J (Jackson Lab, stock: 008462), Pten conditional knockout mice (obtained from Terry Van Dyke [PMC270016]), B6;DBA-Tg(Upk3a-GFP/cre/ERT2)26Amc/J (Jackson Lab, stock: 015855), and FVB.129S6(B6)-Gt(ROSA)26Sortm1(Luc)Kael/J (Jackson Lab, stock: 005125). The resulting UPPL mouse was then backcrossed to C57BL/6 (in house breeding) for 10 generation prior to crossing with the Rosa26-LSL-mApobec3 mice.
Wild animals	N/A
Reporting on sex	Sex was considered in study design. When organoids or cell lines were made, sex was documented.
Field-collected samples	N/A
Ethics oversight	The University of North Carolina at Chapel Hill Institutional Animal Care and Use Committee (IACUC)

Note that full information on the approval of the study protocol must also be provided in the manuscript.
